# 1-(4-{[(*E*)-5-Chloro-2-hy­droxy­benzyl­idene]amino}­phen­yl)ethanone oxime

**DOI:** 10.1107/S1600536810034586

**Published:** 2010-09-04

**Authors:** Li Zhao, Seik Weng Ng

**Affiliations:** a School of Chemical and Biological Engineering, Lanzhou Jiaotong University, Lanzhou 730070, People’s Republic of China; b Department of Chemistry, University of Malaya, 50603 Kuala Lumpur, Malaysia

## Abstract

The title compound, C_15_H_13_ClN_2_O_2_, is an aromatic Schiff base having an aldoxime substituent; the two rings on the azomethine linkage are twisted by 44.4 (1)°. The phenolic H atom is intra­molecularly hydrogen bonded to the azomethine N atom, generating an *S*(6) ring. In the crystal, inversion dimers linked by pairs of O—H⋯N hydrogen bonds occur. The crystal studied was a non-merohedral twin with a 35% minor component.

## Related literature

For background to oxime-type compounds, see: Dong *et al.* (2009 ▶, 2010*b* ▶). For the synthesis, see: Rafiq *et al.* (2008 ▶); Dong *et al.* (2010*a* ▶). For the treatment of non-merohedrally twinned diffraction intensities, see: Spek (2009 ▶). We have reported the crystal structure of one of the first examples of a Schiff base bearing the oxime unit, see: Zhao *et al.* (2009 ▶).
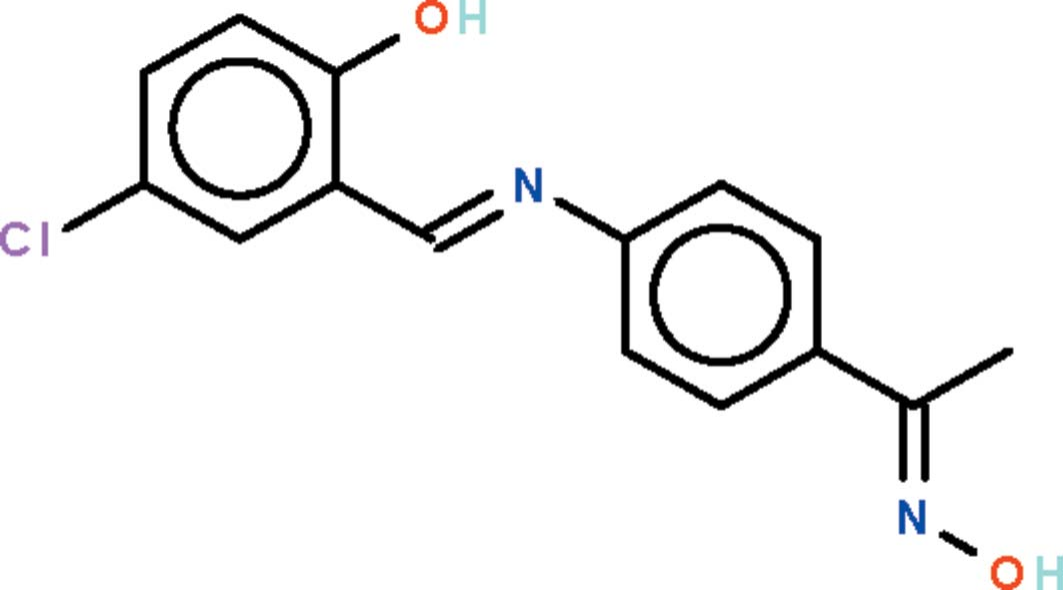


## Experimental

### 

#### Crystal data

C_15_H_13_ClN_2_O_2_
*M* *_r_* = 288.72
Monoclinic, 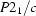

*a* = 15.356 (2) Å
*b* = 14.035 (2) Å
*c* = 6.1124 (6) Å
β = 95.244 (1)°
*V* = 1311.8 (2) Å^3^
*Z* = 4
Mo *K*α radiation
μ = 0.29 mm^−1^
*T* = 293 K
0.40 × 0.10 × 0.05 mm

#### Data collection

Bruker SMART APEX diffractometer
Absorption correction: multi-scan (*SADABS*; Sheldrick, 1996 ▶) *T*_min_ = 0.892, *T*_max_ = 0.986
3689 measured reflections
2971 independent reflections
1552 reflections with *I* > 2σ(*I*)
*R*_int_ = 0.092

#### Refinement

*R*[*F* ^2^ > 2σ(*F* ^2^)] = 0.076
*wR*(*F* ^2^) = 0.239
*S* = 1.02
2970 reflections
192 parameters
2 restraints
H atoms treated by a mixture of independent and constrained refinement
Δρ_max_ = 0.41 e Å^−3^
Δρ_min_ = −0.34 e Å^−3^

### 

Data collection: *SMART* (Bruker, 2001 ▶); cell refinement: *SAINT* (Bruker, 2001 ▶); data reduction: *SAINT*; program(s) used to solve structure: *SHELXS97* (Sheldrick, 2008 ▶); program(s) used to refine structure: *SHELXL97* (Sheldrick, 2008 ▶); molecular graphics: *X-SEED* (Barbour, 2001 ▶); software used to prepare material for publication: *publCIF* (Westrip, 2010 ▶).

## Supplementary Material

Crystal structure: contains datablocks global, I. DOI: 10.1107/S1600536810034586/hg2704sup1.cif

Structure factors: contains datablocks I. DOI: 10.1107/S1600536810034586/hg2704Isup2.hkl

Additional supplementary materials:  crystallographic information; 3D view; checkCIF report

## Figures and Tables

**Table 1:** Hydrogen-bond geometry (Å, °)

*D*—H⋯*A*	*D*—H	H⋯*A*	*D*⋯*A*	*D*—H⋯*A*
O1—H1⋯N1	0.85 (5)	1.81 (3)	2.594 (5)	153 (6)
O2—H2⋯N2^i^	0.86 (5)	2.06 (4)	2.819 (5)	147 (6)

Symmetry code: (i) 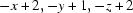.

**Fig. 1.**
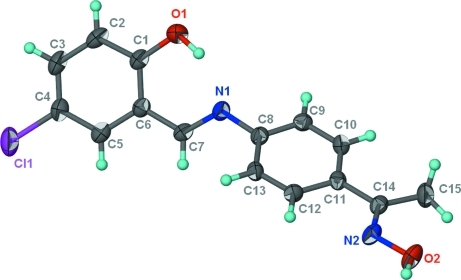
Thermal ellipsoid plot (Barbour, 2001) of C15H13ClN2O at the 50% probability level; hydrogen atoms are drawn as spheres of arbitrary radius.

**Fig. 2.**
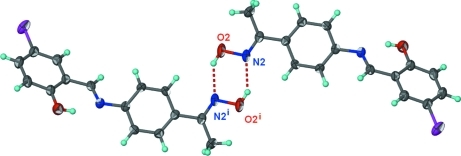
Hydrogen-bonded dimer.

## supplementary crystallographic information

### Comment

Oxime-type compounds are a very important ligands in coordination chemistry (Dong *et al.*, 2010*b*; Dong *et al.*, 2009). 4-Aminophenylethanone oxime is a amino compound having an oxime unit, a unit that can undergo a wide range of transformations. On the other hand, the amino unit lends itself to condensation with carbonyl compounds to yield Schiff bases, yet another class of compounds having an equally wide range of applications. We have reported the crystal structure of one of the first examples of a Schiff base bearing the oxime unit (Zhao *et al.*, 2009). Here we report the synthesis and crystal structure of (*E*)-4-[(1-Hydroxyimino)ethyl]-*N*-(4'-methylbenzylidene)aniline (I), (Fig. 1).

The single-crystal structure of the title compound is built up by discrete C_15_H_13_ClN_2_O_2_ molecules, in which all bond lengths are in normal ranges. Within the molecule, the two rings on the azomethine linkage are twisted by 44.4 (1) °. In the crystal structure, adjacent molecules are connected by an O–H···N_oxime_ hydrogen bond to generate a dimer (Table 1 and Fig. 2).

### Experimental

4-Aminophenylethanone oxime was prepared by 1-(4-aminophenyl)ethanone, hydroxylamine sulfate and sodium acetate (Rafiq *et al.*, 2008; Dong *et al.*, 2010*a*). To an ethanol solution (6 ml) of 4-aminophenylethanone oxime (152.1 mg, 1.00 mmol) was added dropwise an ethanol solution (6 ml) of 5-chlorosalicylaldehyde (159.2 mg, 1.00 mmol). The mixture solution was stirred at 328 K for 5 h. After cooling to room temperature, the precipitate was filtered off, and washed successively three times with ethanol. The product was dried *in vacuo* and purified by recrystallization from ethanol to yield 266.5 mg (Yield, 92.3%) of solid; m.p. 484–486 K. Pale-yellow needle-like single crystals suitable for X-ray diffraction studies were obtained by slow evaporation from a mixed solution of ethyl acetate-chloroform (3:2) of (I) at room temperature for about four weeks. Anal. Calcd. for C_15_H_13_ClN_2_O_2_: C, 62.40; H, 4.54; N, 9.70; Found: C, 62.22; H, 4.50; N, 9.85.

### Refinement

Carbon-bound H-atoms were placed in calculated positions (C—H 0.95 to 0.96 Å) and were included in the refinement in the riding model approximation, with U(H) set to 1.2 to 1.5U(C).

The hydroxy H-atoms were located in a difference Fourier map, and were refined with a distance restraint of O–H of 0.85±0.01 Å; their temperature factors were freely refined.

The crystal is a non-merohedral twin; the diffraction intensities were separated into two domains by using *PLATON* (Spek, 2009); the minor twin component was 35%.

The somewhat large weighting scheme is probably the consequence of the twinning. Lowering the 2θ limit to 50 ° leads to a marginally better refinement but the weighting scheme is identical.

### Crystal data

**Table:**

C_15_H_13_ClN_2_O_2_	*F*(000) = 600
*M**_r_* = 288.72	*D*_x_ = 1.462 Mg m^−^^3^
Monoclinic, *P*2_1_/*c*	Mo *K*α radiation, λ = 0.71073 Å
Hall symbol: -P 2ybc	Cell parameters from 1371 reflections
*a* = 15.356 (2) Å	θ = 2.7–24.9°
*b* = 14.035 (2) Å	µ = 0.29 mm^−^^1^
*c* = 6.1124 (6) Å	*T* = 293 K
β = 95.244 (1)°	Needle-like, yellow
*V* = 1311.8 (2) Å^3^	0.40 × 0.10 × 0.05 mm
*Z* = 4	

### Data collection

**Table:**

Bruker SMART APEX diffractometer	2971 independent reflections
Radiation source: fine-focus sealed tube	1552 reflections with *I* > 2σ(*I*)
graphite	*R*_int_ = 0.092
φ and ω scans	θ_max_ = 27.5°, θ_min_ = 2.0°
Absorption correction: multi-scan (*SADABS*; Sheldrick, 1996)	*h* = −19→19
*T*_min_ = 0.892, *T*_max_ = 0.986	*k* = −18→18
3689 measured reflections	*l* = −2→7

### Refinement

**Table:**

Refinement on *F*^2^	Primary atom site location: structure-invariant direct methods
Least-squares matrix: full	Secondary atom site location: difference Fourier map
*R*[*F*^2^ > 2σ(*F*^2^)] = 0.076	Hydrogen site location: inferred from neighbouring sites
*wR*(*F*^2^) = 0.239	H atoms treated by a mixture of independent and constrained refinement
*S* = 1.02	*w* = 1/[σ^2^(*F*_o_^2^) + (0.1159*P*)^2^] where *P* = (*F*_o_^2^ + 2*F*_c_^2^)/3
2970 reflections	(Δ/σ)_max_ = 0.001
192 parameters	Δρ_max_ = 0.41 e Å^−^^3^
2 restraints	Δρ_min_ = −0.34 e Å^−^^3^

### Fractional atomic coordinates and isotropic or equivalent isotropic displacement parameters (Å^2^)

**Table:**

	*x*	*y*	*z*	*U*_iso_*/*U*_eq_	
Cl1	0.26367 (8)	0.34104 (10)	0.2064 (3)	0.0716 (5)	
N1	0.6685 (2)	0.3635 (2)	0.1036 (6)	0.0378 (9)	
O1	0.5752 (2)	0.4086 (3)	−0.2569 (6)	0.0543 (9)	
N2	0.9958 (2)	0.4344 (2)	0.8002 (6)	0.0444 (10)	
O2	1.0777 (2)	0.4341 (2)	0.9205 (7)	0.0565 (10)	
C1	0.5046 (3)	0.3912 (3)	−0.1470 (8)	0.0395 (11)	
C2	0.4222 (3)	0.4095 (3)	−0.2484 (8)	0.0454 (11)	
H2A	0.4164	0.4333	−0.3910	0.054*	
C3	0.3485 (3)	0.3930 (3)	−0.1418 (9)	0.0481 (12)	
H3	0.2933	0.4051	−0.2124	0.058*	
C4	0.3566 (3)	0.3589 (3)	0.0678 (9)	0.0421 (11)	
C5	0.4378 (3)	0.3407 (3)	0.1758 (8)	0.0407 (10)	
H5	0.4424	0.3178	0.3192	0.049*	
C6	0.5131 (3)	0.3569 (3)	0.0687 (7)	0.0353 (10)	
C7	0.5974 (3)	0.3471 (3)	0.1921 (7)	0.0359 (10)	
H7	0.6002	0.3285	0.3386	0.043*	
C8	0.7485 (2)	0.3677 (3)	0.2391 (7)	0.0350 (10)	
C9	0.8234 (3)	0.3372 (3)	0.1531 (7)	0.0359 (10)	
H9	0.8206	0.3124	0.0116	0.043*	
C10	0.9030 (3)	0.3438 (3)	0.2787 (8)	0.0368 (10)	
H10	0.9531	0.3219	0.2201	0.044*	
C11	0.9104 (2)	0.3813 (3)	0.4848 (7)	0.0327 (10)	
C12	0.8338 (2)	0.4151 (3)	0.5670 (7)	0.0368 (10)	
H12	0.8368	0.4427	0.7058	0.044*	
C13	0.7546 (3)	0.4079 (3)	0.4454 (7)	0.0374 (10)	
H13	0.7044	0.4304	0.5027	0.045*	
C14	0.9956 (3)	0.3880 (3)	0.6196 (8)	0.0363 (10)	
C15	1.0748 (3)	0.3417 (3)	0.5426 (9)	0.0524 (13)	
H15A	1.1216	0.3436	0.6580	0.079*	
H15B	1.0921	0.3752	0.4165	0.079*	
H15C	1.0616	0.2767	0.5037	0.079*	
H1	0.619 (3)	0.395 (4)	−0.166 (8)	0.10 (2)*	
H2	1.076 (4)	0.471 (4)	1.031 (8)	0.12 (3)*	

### Atomic displacement parameters (Å^2^)

**Table:**

	*U*^11^	*U*^22^	*U*^33^	*U*^12^	*U*^13^	*U*^23^
Cl1	0.0347 (6)	0.0879 (10)	0.0928 (13)	0.0041 (6)	0.0083 (7)	0.0070 (9)
N1	0.0316 (19)	0.0419 (19)	0.039 (2)	0.0017 (14)	−0.0015 (17)	−0.0003 (16)
O1	0.048 (2)	0.075 (2)	0.040 (2)	0.0013 (17)	0.0039 (18)	0.0068 (18)
N2	0.0351 (19)	0.050 (2)	0.045 (3)	−0.0027 (16)	−0.0121 (18)	0.0007 (19)
O2	0.0406 (17)	0.061 (2)	0.064 (3)	0.0004 (15)	−0.0211 (17)	−0.004 (2)
C1	0.037 (2)	0.038 (2)	0.041 (3)	0.0029 (18)	−0.005 (2)	−0.003 (2)
C2	0.050 (3)	0.041 (2)	0.043 (3)	0.006 (2)	−0.014 (2)	−0.001 (2)
C3	0.037 (2)	0.045 (3)	0.059 (4)	0.0054 (19)	−0.011 (2)	−0.005 (2)
C4	0.033 (2)	0.039 (2)	0.053 (3)	0.0005 (17)	−0.001 (2)	−0.005 (2)
C5	0.040 (2)	0.037 (2)	0.044 (3)	0.0021 (18)	−0.004 (2)	−0.002 (2)
C6	0.032 (2)	0.036 (2)	0.037 (3)	−0.0014 (17)	−0.0029 (19)	−0.0036 (19)
C7	0.039 (2)	0.034 (2)	0.033 (2)	0.0015 (17)	−0.004 (2)	−0.0011 (19)
C8	0.032 (2)	0.035 (2)	0.037 (3)	−0.0023 (16)	−0.0002 (19)	0.0018 (19)
C9	0.039 (2)	0.038 (2)	0.030 (2)	−0.0001 (17)	0.0020 (19)	−0.0032 (18)
C10	0.030 (2)	0.039 (2)	0.042 (3)	0.0003 (17)	0.005 (2)	−0.002 (2)
C11	0.028 (2)	0.030 (2)	0.040 (3)	−0.0040 (15)	0.0033 (19)	0.0008 (18)
C12	0.037 (2)	0.043 (2)	0.030 (2)	−0.0021 (18)	−0.0006 (19)	−0.0060 (19)
C13	0.029 (2)	0.046 (2)	0.037 (3)	0.0025 (17)	0.0045 (19)	−0.003 (2)
C14	0.032 (2)	0.033 (2)	0.042 (3)	−0.0031 (17)	−0.002 (2)	0.005 (2)
C15	0.031 (2)	0.066 (3)	0.060 (3)	0.003 (2)	0.005 (2)	−0.005 (3)

### Geometric parameters (Å, °)

**Table:**

Cl1—C4	1.743 (5)	C6—C7	1.444 (6)
N1—C7	1.282 (5)	C7—H7	0.9300
N1—C8	1.419 (5)	C8—C13	1.376 (6)
O1—C1	1.348 (5)	C8—C9	1.375 (5)
O1—H1	0.85 (5)	C9—C10	1.387 (6)
N2—C14	1.281 (6)	C9—H9	0.9300
N2—O2	1.398 (4)	C10—C11	1.360 (6)
O2—H2	0.86 (5)	C10—H10	0.9300
C1—C2	1.382 (6)	C11—C12	1.403 (5)
C1—C6	1.398 (6)	C11—C14	1.484 (5)
C2—C3	1.376 (6)	C12—C13	1.370 (5)
C2—H2A	0.9300	C12—H12	0.9300
C3—C4	1.363 (7)	C13—H13	0.9300
C3—H3	0.9300	C14—C15	1.492 (6)
C4—C5	1.379 (6)	C15—H15A	0.9600
C5—C6	1.400 (6)	C15—H15B	0.9600
C5—H5	0.9300	C15—H15C	0.9600
			
C7—N1—C8	119.1 (4)	C13—C8—N1	122.3 (4)
C1—O1—H1	104 (4)	C9—C8—N1	118.3 (4)
C14—N2—O2	112.6 (4)	C8—C9—C10	119.6 (4)
N2—O2—H2	109 (5)	C8—C9—H9	120.2
O1—C1—C2	119.2 (4)	C10—C9—H9	120.2
O1—C1—C6	121.5 (4)	C11—C10—C9	122.2 (4)
C2—C1—C6	119.3 (4)	C11—C10—H10	118.9
C3—C2—C1	121.1 (5)	C9—C10—H10	118.9
C3—C2—H2A	119.5	C10—C11—C12	117.4 (4)
C1—C2—H2A	119.5	C10—C11—C14	122.2 (4)
C4—C3—C2	119.7 (4)	C12—C11—C14	120.4 (4)
C4—C3—H3	120.2	C13—C12—C11	120.9 (4)
C2—C3—H3	120.2	C13—C12—H12	119.6
C3—C4—C5	121.1 (4)	C11—C12—H12	119.6
C3—C4—Cl1	119.9 (4)	C12—C13—C8	120.6 (4)
C5—C4—Cl1	119.0 (4)	C12—C13—H13	119.7
C4—C5—C6	119.7 (5)	C8—C13—H13	119.7
C4—C5—H5	120.1	N2—C14—C11	116.3 (4)
C6—C5—H5	120.1	N2—C14—C15	123.7 (4)
C1—C6—C5	119.2 (4)	C11—C14—C15	120.0 (4)
C1—C6—C7	121.8 (4)	C14—C15—H15A	109.5
C5—C6—C7	118.7 (4)	C14—C15—H15B	109.5
N1—C7—C6	121.3 (4)	H15A—C15—H15B	109.5
N1—C7—H7	119.4	C14—C15—H15C	109.5
C6—C7—H7	119.4	H15A—C15—H15C	109.5
C13—C8—C9	119.2 (4)	H15B—C15—H15C	109.5
			
O1—C1—C2—C3	−179.8 (4)	C7—N1—C8—C9	147.4 (4)
C6—C1—C2—C3	−1.0 (6)	C13—C8—C9—C10	2.7 (6)
C1—C2—C3—C4	0.5 (6)	N1—C8—C9—C10	177.6 (4)
C2—C3—C4—C5	0.2 (7)	C8—C9—C10—C11	−1.2 (6)
C2—C3—C4—Cl1	178.2 (3)	C9—C10—C11—C12	−1.0 (6)
C3—C4—C5—C6	−0.4 (6)	C9—C10—C11—C14	179.8 (4)
Cl1—C4—C5—C6	−178.4 (3)	C10—C11—C12—C13	1.7 (6)
O1—C1—C6—C5	179.6 (4)	C14—C11—C12—C13	−179.0 (4)
C2—C1—C6—C5	0.8 (6)	C11—C12—C13—C8	−0.3 (7)
O1—C1—C6—C7	6.1 (6)	C9—C8—C13—C12	−1.9 (6)
C2—C1—C6—C7	−172.6 (4)	N1—C8—C13—C12	−176.6 (4)
C4—C5—C6—C1	−0.1 (6)	O2—N2—C14—C11	177.7 (3)
C4—C5—C6—C7	173.6 (4)	O2—N2—C14—C15	−1.9 (6)
C8—N1—C7—C6	170.1 (3)	C10—C11—C14—N2	172.3 (4)
C1—C6—C7—N1	−4.7 (6)	C12—C11—C14—N2	−7.0 (6)
C5—C6—C7—N1	−178.2 (3)	C10—C11—C14—C15	−8.1 (6)
C7—N1—C8—C13	−37.9 (6)	C12—C11—C14—C15	172.7 (4)

### Hydrogen-bond geometry (Å, °)

**Table:**

*D*—H···*A*	*D*—H	H···*A*	*D*···*A*	*D*—H···*A*
O1—H1···N1	0.85 (5)	1.81 (3)	2.594 (5)	153 (6)
O2—H2···N2^i^	0.86 (5)	2.06 (4)	2.819 (5)	147 (6)

Symmetry codes: (i) −*x*+2, −*y*+1, −*z*+2.
